# *Platycodon grandiflorum*: from phytochemical diversity to polysaccharides' prominent bioactivities and edible-medicinal applications

**DOI:** 10.3389/fnut.2025.1742082

**Published:** 2026-01-12

**Authors:** Mengnan Chen, Wenfeng Wei, Zhuang Wang, Jinhai Huo, Weiming Wang

**Affiliations:** 1 Harbin University of Commerce, Harbin, China; 2 Heilongjiang Academy of Traditional Chinese Medicine, Harbin, China

**Keywords:** edible-medicinal application, functional food application, green extraction technology, immunomodulatory mechanism, phytochemistry, quality control, structural modification

## Abstract

*Platycodon grandiflorum* (*P. grandiflorum*), a plant traditionally recognized for its dual application in medicine and food, is extensively utilized in East Asian traditional practices and culinary arts. This review consolidates recent findings on the phytochemical composition of *P. grandiflorum*, with an emphasis on the bioactivities, applications, and quality control of its key constituent—*Platycodon grandiflorum* polysaccharides (PGPs). The plant encompasses a variety of bioactive compounds, including saponins (with platycodin D as a marker), volatile oils, flavonoids, and PGPs, which are heteropolysaccharides predominantly composed of arabinose, galactose, and glucose. Notably, PGPs are distinguished by their non-toxicity, biocompatibility, and extensive therapeutic potential. Ultrasound-assisted extraction (UAE) methods have been shown to yield 22%−37% more PGPs than conventional techniques. These polysaccharides demonstrate significant immunomodulatory, antioxidant, metabolic regulatory, antitumor, and hepatorenal protective properties. While PGPs find utility in functional foods, pharmaceuticals, and cosmetics, challenges such as limited solubility persist. A robust quality control framework for PGPs is recommended. Future research endeavors should focus on strategic structural modifications, clinical assessments, and sustainable extraction methodologies to further its industrial applicability.

## 1 Introduction

*Platycodon grandiflorum* (Jacq.) A. DC. (balloon flower), a representative “homology of medicine and food” plant, has been utilized for centuries in East Asian traditional medicine and dietary practices due to its potential health benefits (1). Modern scientific research has progressively elucidated the chemical basis underlying its medicinal and nutritional properties, establishing it as a focal point in phytomedicine and functional food development. Early studies on *P. grandiflorum* primarily focused on its saponin components, with platycodin D serving as the characteristic marker—this compound is designated as the identification index for *P. grandiflorum* raw materials and decoction pieces in the Pharmacopoeia of the People's Republic of China (2020 Edition, Volume I) (2, 3). While saponins—particularly platycodin D—are recognized as the hallmark bioactive components of Platycodon grandiflorus and listed in official pharmacopoeias, the plant-derived polysaccharides have recently emerged as a research hotspot. This growing attention stems from their superior safety profile, marked by inherent non-toxicity and excellent biocompatibility, as well as their diverse biological activities; notably, their immunomodulatory efficacy has been shown to complement or even surpass that of saponins (4–6).

**Figure 1:**
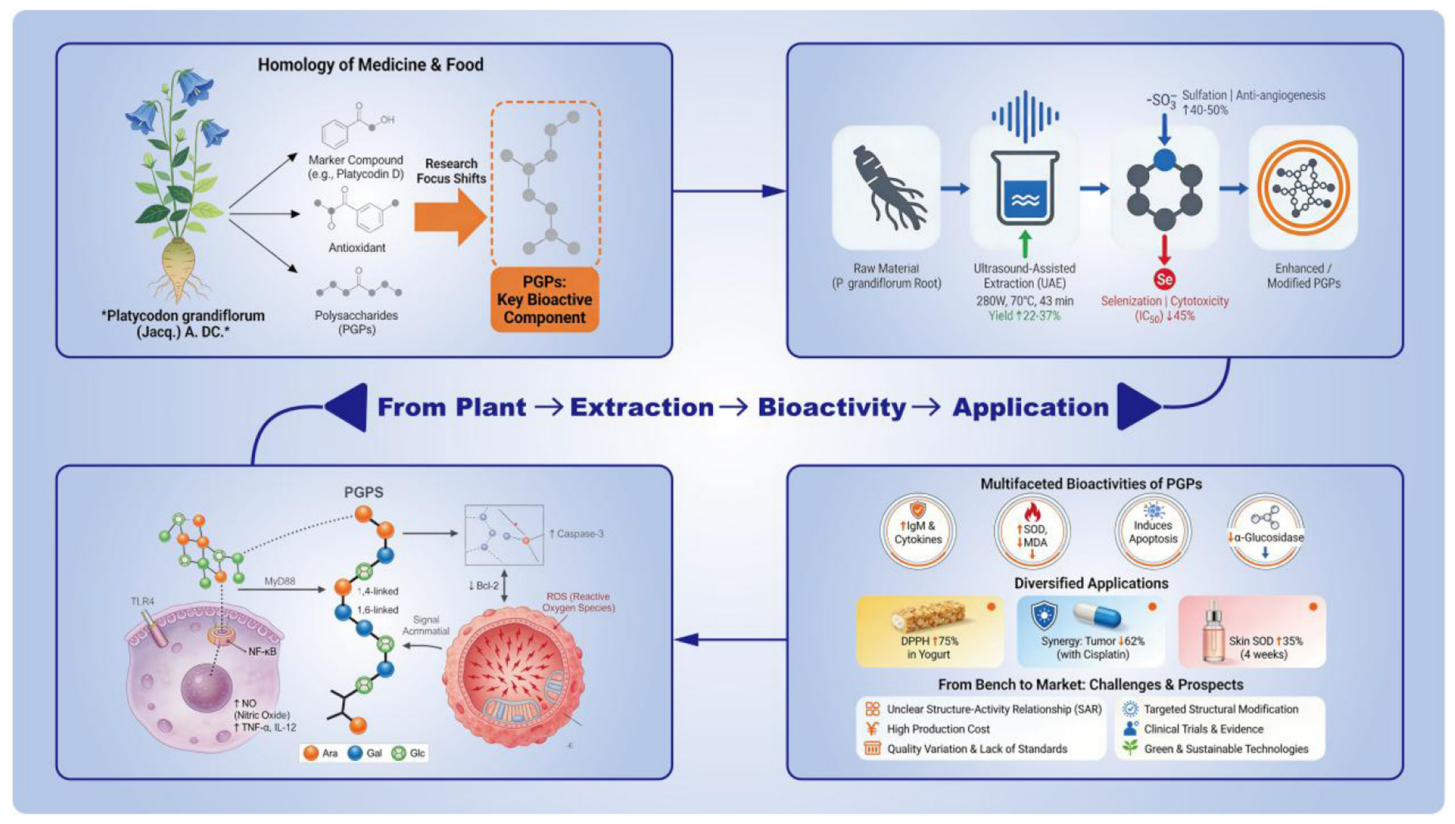
*Platycodon grandiflorum* polysaccharides (PGPs): from extraction, structural modification to bioactivities and industrial applications.

Advances in extraction and modification technologies have greatly promoted the research of *P. grandiflorum*. The traditional hot water extraction (HWE) of polysaccharides (PGPs) has low yield and may cause structural damage, while UAE can increase the yield of PGPs by 22%−37% compared to HWE under controlled conditions (4, 7). It is important to note that these values are optimal laboratory results and may be affected by factors such as plant age, particle size, and equipment calibration. Chemical modifications such as sulfation and selenization can further enhance the bioactivity of PGPs: sulfated derivatives exhibit 40%−50% higher anti-angiogenic activity, and selenized derivatives show significantly improved antitumor effects (5, 8). Despite these technological advancements, several challenges remain unresolved. The structure-activity relationships (SARs) governing the functions of PGPs remain unclear, with correlations between structural parameters (e.g., branching degree, glycosidic linkage types) and biological activities (e.g., immunomodulation, antitumor effects) yet to be fully elucidated (9). Current studies on bioactivities mainly rely on *in vitro* cellular or animal models, lacking sufficient clinical evidence to support their efficacy and safety in humans (10, 11). Additionally, industrial applications face obstacles including poor aqueous solubility of high-molecular-weight PGPs (>50 kDa), high costs associated with advanced extraction methods, and batch-to-batch variability due to differences in geographical origin and processing techniques (7, 12). To visually synthesize the cumulative progress, existing challenges and multi-dimensional research framework of PGPs as discussed above, Figure 1 provides a panoramic view of the PGP research landscape by integrating core information including extraction parameters, modification effects, key bioactivities and application scenarios.

To address these gaps, this review systematically summarizes the phytochemical composition of *P. grandiflorum* with a focus on PGPs—including their extraction and purification, structural characteristics, biological activities and mechanisms, edible-medicinal applications, and quality control. It also proposes future research directions to promote PGP industrialization, providing a theoretical basis for the scientific utilization of this valuable “homology of medicine and food” resource.

## 2 Chemical constituents of *Platycodon grandiflorum*

### 2.1 Saponins

Among these components, saponins represent a diverse group of natural glycosides, characterized by triterpenoid or steroidal aglycones linked to one or more sugar chains (1), exhibiting unique physicochemical properties (e.g., soap-like foaming in water and micelle formation) that affect the absorption and excretion of compounds in the body (13). *P. grandiflorum* is characterized by “platycosides,” which are triterpenoid saponins with two sugar chains predominantly concentrated in its roots (1). The saponin content in *P. grandiflorum* is affected by factors such as growth environment (14) and processing methods (15); fermentation and aging can simultaneously increase saponin content while reducing bitterness (16). To date, over 80 saponin components have been isolated from *P. grandiflorum* (17), among which platycodin A, platycodin D, and platycodin D3 demonstrate significant immunomodulatory, antitumor, anti-inflammatory, and hepatoprotective activities (2). Notably, platycodin D serves as the characteristic component of *P. grandiflorum* and its processed products and has been designated as an identification index for *P. grandiflorum* raw materials and decoction pieces in the Pharmacopoeia of the People's Republic of China (2020 Edition, Volume I). Therefore, investigating changes in platycodin D content before and after processing holds critical importance for enhancing quality standards of processed *P. grandiflorum* products. As the official identification marker for *P. grandiflorum* raw materials and decoction pieces in the Chinese Pharmacopoeia (2020 Edition, Volume I), monitoring platycodin D content during processing is essential for product quality control (2).

While saponins have long been the focus of *P. grandiflorum* research, the discovery of PGPs has broadened the potential applications of this plant. Unlike saponins, which may exhibit mild cytotoxicity at high doses, PGPs are non-toxic and biocompatible, and they possess a broader spectrum of bioactivities (e.g., gut microbiota regulation and metabolic modulation) that complement the effects mediated by saponins (18, 19). This complementarity underscores the importance of conducting integrated studies on both components to fully harness the medicinal and nutritional value of *P. grandiflorum*.

### 2.2 Flavonoids

To date, more than 10 types of flavonoid components have been isolated and identified from *P. grandiflorum*, mainly including dihydroflavones, flavones, and their derivatives. Japanese researchers first isolated platyconin (a caffeoyl rutinol glycoside) from *P. grandiflorum* (14), and subsequently identified five flavonoid components, including (2R,3R)-taxifolin, quercetin-7-*O*-glucoside, and quercetin-7-*O*-rutinoside (18, 20). Polish researchers isolated two flavonoid compounds, luteolin and apigenin, from the aerial parts of *P. grandiflorum* (21). Additionally, flavoplatycoside, grandoside (3-methyl-1-butanol glycoside), and other flavonoids have been detected in *P. grandiflorum* (22).

### 2.3 *Platycodon grandiflorum* polysaccharides (PGPs)

#### 2.3.1 Structural characteristics of PGPs

##### 2.3.1.1 Monosaccharide composition

PGPs are heteropolysaccharides primarily composed of arabinose (Ara), galactose (Gal), and glucose (Glc), with trace rhamnose (Rha) and galacturonic acid (GalA) (12). The monosaccharide molar ratio varies with *P. grandiflorum* origin and extraction method: Northeast China (Heilongjiang): Ara:Gal:Glc = 1.42:1.00:0.85 (4); South Korea (Gyeonggi): Ara:Gal:Glc = 1.21:1.00:0.92 (23) Japan (Hokkaido): Ara:Gal:Glc = 1.53:1.00:0.78 (20). Acidic PGPs (containing GalA) account for 30%−40% of total PGPs and exhibit stronger antioxidant activity than neutral PGPs. This enhanced activity is attributed to the electron-donating effect of carboxyl groups (-COOH) in GalA, which can scavenge free radicals by donating hydrogen atoms (6).

##### 2.3.1.2 Glycosidic linkage and backbone structure

Advanced techniques (^1^H-NMR, ^13^C-NMR, and methylation) have elucidated the structures of PGPs, with the structural details of typical PGP fractions—including backbone and branching features—presented in Table 1. The backbone of most PGPs is composed of 1,4-linked galactopyranose (Galp) and/or 1,6-linked Galp with branching mainly at O-3 or O-6 positions of sugar residues. For example, the branched PGP fraction PGAW1 has a backbone of 1,4- and 1,6-linked Galp with Ara residues attached to the O-3 position of 1,6-linked Galp (19). This branched structure gives 2–3 times stronger immunomodulatory activity than linear PGPs (e.g., PGPSt which has an unbranched 1,4-linked Galp backbone). The mechanism underlying this difference is that the branch sites increase the binding affinity to immune cell receptors (e.g., Toll-like receptor 4, TLR4), activating downstream signaling pathways to enhance immune cell proliferation and cytokine secretion (24).

**Table 1:** Structural characteristics and representative bioactivities of selected PGP fractions.

**PGP fraction**	**Molecular weight (kDa)**	**Backbone structure**	**Branching features**	**Bioactivity highlight**	**Reference**
PGAW1	6.5	1,4-linked Galp + 1,6-linked Galp	Ara residues at O-3 of 1,6-linked Galp	Immunomodulation (↑B cell proliferation)	(19)
PGPSt	6.4	1,4-linked Galp	No branching	Immunomodulation (activates chicken peritoneal macrophages)	(24)
PGP40-2B	12.3	1,4-linked Glcp	Gal residues at O-6 of 1,4-linked Glcp	Antitumor (↓A549 cell viability)	(32)

##### 2.3.1.3 Structural modification

To enhance PGPs' bioactivity and solubility, chemical and biological modifications are widely used: sulfation: using chlorosulfonic acid-pyridine (1:3, v/v) at 60 °C for 2 h, the degree of substitution (DS) reaches 1.2–1.8. Sulfated PGPs exhibit 40%−50% stronger anti-angiogenic activity by inhibiting human microvascular endothelial cell (HMVEC) tube formation (19). Selenization: sodium selenite-citric acid method (selenium content: 0.08%−0.15%) enhances antitumor activity—selenized PGPs reduce HepG2 cell viability with an IC50 of 15.6 μM, compared to 28.3 μM for natural PGPs (8). Fermentation: *Lactobacillus plantarum* fermentation reduces PGPs' molecular weight from 12.3 to 6.8 kDa, increasing their solubility by 60% and improving gut microbiota regulatory activity (25).

#### 2.3.2 Extraction and purification of PGPs

Extraction serves as the foundational step in polysaccharide glycoside (PGP) research, where both efficiency and structural integrity are paramount. Table 2 presents a comparative analysis of four mainstream extraction techniques for PGPs, summarizes their optimal parameters, yield, advantages, and disadvantages, and identifies UAE as particularly suitable for industrial implementation. Li et al. (15) established optimal UAE parameters for PGPs: ultrasonic power at 280 W, extraction temperature at 70 °C, and duration of 43 min. These conditions yielded 15.3% PGP (dry basis) while preserving glycosidic linkage integrity—a critical requirement for maintaining bioactivity given these bonds constitute the functional framework of polysaccharides. Enzymatic pretreatment enhances UAE efficacy; specifically, combining UAE with cellulase-xylanase mixture (1:1 mass ratio, 0.8% dosage relative to raw material) increases PGP yield by 10%−15% (7). This enzymatic approach disrupts plant cell wall matrices, exposing intracellular polysaccharides and facilitating their release during ultrasonic cavitation. Crude PGP extracts require purification to remove contaminants (proteins, pigments, low-molecular-weight sugars) that could compromise bioactivity and application safety through three sequential steps: (1) Protein elimination via Sevag method (chloroform:n-butanol = 4:1, v/v), which achieves 85%−92% protein removal (4) by selectively precipitating proteins through solvent interaction while retaining PGPs in the aqueous phase; (2) depigmentation using D101 macroporous resin adsorption, removing >90% pigments with >88% PGP recovery (12) through hydrophobic interactions between resin groups and pigment molecules while excluding hydrophilic polysaccharides; and (3) fractionation employing Sephadex G-100 or DEAE-cellulose column chromatography to separate PGPs into homogeneous fractions (neutral and acidic PGPs) (19) based on molecular weight and charge differentials. Xu et al. (5) developed an integrated “UAE + membrane separation” process, wherein a 50 kDa ultrafiltration membrane retains PGPs and removes small-molecule impurities after UAE extraction, followed by Sephadex G-100 purification. This method achieves >90% PGP purity with a total recovery rate of 78.5%, effectively balancing efficiency and product quality.

**Table 2:** Comparative analysis of extraction techniques for *Platycodon grandiflorum* polysaccharides (PGPs).

**Extraction method**	**Optimal parameters**	**Yield (dry basis)**	**Advantages**	**Disadvantages**	**Reference**
Microwave-assisted extraction	600 W, 5–10 min, 1:15 (w/v)	11.8%−14.5%	Ultra-fast, energy-saving	Uneven heating (local overheating)	(3)
Hot water extraction (HWE)	80–90 °C, 2–4 h, 1:20 (w/v) liquid-solid ratio	7.62%−9.8%	Low cost, simple operation	Long time, high temperature (destroys partial structure)	(4)
Ultrasound-assisted extraction (UAE)	150–300 W, 70 °C, 20–45 min, 1:20 (w/v)	12.01%−15.3%	High yield (↑22%−37% vs. HWE), short time	High equipment cost	(7)
Enzyme-assisted extraction	Cellulase (0.6%−1.0%), 50 °C, 1–2 h	10.5%−13.2%	Mild conditions, preserves bioactivity	Enzyme cost, potential protein contamination	(41)

#### 2.3.3 Bioactivities of PGPs and molecular mechanisms

##### 2.3.3.1 Immunomodulatory activity

PGPs can significantly regulate both innate and adaptive immune responses (26, 27). *in vitro* and *in vivo* studies have confirmed that PGPs can activate immune cells such as B cells and macrophages (26). Han et al. (26) found that polysaccharides isolated from *P. grandiflorum* significantly promoted the production of polyclonal IgM antibodies and the proliferation of B cells, as well as activated the transcription of inducible nitric oxide synthase (iNOS) and the production of nitric oxide (NO) in macrophages. Additionally, PGPs can induce the maturation of dendritic cells (DCs), laying a crucial foundation for initiating adaptive immune responses (27). Park et al. (27) demonstrated that PGPs induced the phenotypic maturation of DCs, upregulated the expression of cell surface co-stimulatory molecules (CD40, CD80, CD86) and antigen-presenting molecules (MHC-I/II), and promoted the secretion of pro-inflammatory cytokines such as IL-12, TNF-α, and IL-1β. The immunomodulatory activity of PGPs is mainly mediated through the Toll-like receptor 4 (TLR4) signaling pathway (27, 28). Yoon et al. (28) clarified that PGPs induce NO production in macrophages via a TLR4-dependent mechanism, involving the activation of NF-κB signaling. These findings suggest that PGPs have potential as immunostimulants or adjuvants to enhance the body's immune response against pathogens and diseases. Compared to other plant-derived polysaccharides, such as Astragalus polysaccharide and lentinan, PGPs have a stronger activation effect on B cells and dendritic cells, which may be related to their unique branched structure and specific glycosidic linkage composition. For example, Astragalus polysaccharide mainly enhances macrophage phagocytosis (29), while PGPs have broad immunomodulatory effects on various immune cell types, indicating that they can play a complementary role in the formulation of immune regulation.

##### 2.3.3.2 Antioxidant activity

PGPs possess strong antioxidant capacity and can protect cells from oxidative damage induced by free radicals (6, 30). Li et al. (4) showed that PGPs extracted by UAE and HWE both exhibited specific antioxidant activities, including effectively scavenging DPPH radicals, ABTS radicals, and hydroxyl radicals *in vitro*, as well as alleviating H_2_O_2_-induced damage in HepG2 cells. Sheng et al. (6) reported that a selenium-containing polysaccharide (PGP1) isolated from *P. grandiflorum* protected PC12 cells from H_2_O_2_-induced oxidative damage by increasing superoxide dismutase (SOD) activity and decreasing malondialdehyde (MDA) levels. The antioxidant mechanism of PGPs mainly relies on their free radical scavenging ability and oxidative stress alleviation, providing potential for preventing or delaying the onset of chronic diseases. The antioxidant capacity of PGPs exhibits structural dependency, as acidic PGPs containing GalA residues demonstrate enhanced radical scavenging activity relative to neutral PGPs. This phenomenon may be attributed to the electron-donating properties of carboxyl groups present in the acidic fractions. Notably, this mechanism differs from that observed in *Ganoderma lucidum* polysaccharides, where β-glucan structures primarily contribute to antioxidant effects (31). Furthermore, PGPs obtained through UAE maintain superior antioxidant activity compared to those extracted via conventional HWE, highlighting the critical influence of extraction methodologies on preserving functional bioactive components.

##### 2.3.3.3 Antitumor activity

Several studies have confirmed that PGPs exhibit promising antitumor potential in both *in vitro* and *in vivo* models (8, 9, 30, 32). Zhang et al. (8) found that selenized glucomannan (Se-PGP40-1) derived from *P. grandiflorum* significantly inhibited tumor proliferation and migration by inducing tumor cell apoptosis and blocking angiogenesis in cell and zebrafish models. A polysaccharide fraction (PGP40-2B) isolated by Liu et al. (32) showed *in vivo* antitumor effects in a zebrafish model, possibly by targeting the VEGF and PD-1 pathways to inhibit angiogenesis and activate immune responses. Lee et al. (30) demonstrated that organic extracts from *P. grandiflorum* roots exhibited cytotoxicity against human cancer cell lines, with the anticancer activity partially showing the typical UV absorption spectrum of polyacetylenes. These studies indicate that PGPs have the potential to be developed into novel antitumor drugs. PGPs exert antitumor effects through multiple mechanisms, including the induction of apoptosis (33), inhibition of angiogenesis, and immune activation (34). Selenized PGPs exhibit enhanced cytotoxicity relative to their native counterparts, with a mechanistic shift from immune modulation toward direct ROS induction. This dual-action strategy—combining immune enhancement with direct tumor cell targeting—positions PGPs as versatile candidates for adjunctive cancer therapy, in contrast to single-mechanism agents such as paclitaxel or doxorubicin.

##### 2.3.3.4 Other bioactivities

In addition to the above core activities, PGPs exhibit various other beneficial effects. Li et al. (4) confirmed that PGPs have strong inhibitory activity against α-amylase and α-glucosidase, and can enhance glucose uptake and glycogen content in insulin-resistant HepG2 cells. These results suggest that PGPs can exert hypoglycemic effects by inhibiting carbohydrate hydrolysis and improving insulin sensitivity, possibly without stimulating insulin secretion (35). PGPs have shown protective effects against liver injury in experimental models (22, 32). PGPs have also been found to inhibit apoptosis in certain cell types, indicating their potential role in cell survival and tissue protection (9, 12). By suppressing the cytokine storm, PGPs can significantly alleviate inflammation and cytokine storm syndrome in mice with acute lung injury (ALI) by neutralizing multiple pro-inflammatory cytokines (36, 37). PGPs have been shown to improve obesity induced by a high-fat diet by regulating the gut microbiota (38). The multi-target bioactivity profile of PGPs—spanning metabolic regulation, hepatoprotection, anti-inflammatory, and gut microbiota modulation—highlights their potential as multi-functional therapeutic agents. Unlike single-component drugs, PGPs offer a holistic approach to disease management, particularly in metabolic syndrome and inflammatory conditions.

##### 2.3.3.5 Synergistic effects of structural modification

Chemical modification can significantly enhance PGP bioactivity and expand its application scope (12), with sulfation and selenization being the most widely used methods (8, 19). Sulfation is typically performed using a chlorosulfonic acid-pyridine solution (1:3, v/v) at 60 °C for 2 h, resulting in derivatives with a degree of substitution (DS) ranging from 1.2 to 1.8. These derivatives exhibit 40%−50% greater anti-angiogenic activity than native PGPs (19). The mechanism involves negatively charged sulfate groups (-$SO_{3}^{-}$) binding to positively charged amino acid residues on vascular endothelial growth factor receptors (VEGFRs) of human microvascular endothelial cells (HMVECs), thereby inhibiting VEGFR-mediated signaling and blocking tube formation, a critical step in tumor angiogenesis. In contrast, selenization employs a distinct mechanism to achieve potent antitumor effects. Selenized PGPs are synthesized via the sodium selenite-citric acid method (8). This modification markedly improves cytotoxicity; for example, against HepG2 cells, the IC_50_ decreases from 28.3 μM (native PGPs) to 15.6 μM (selenized PGPs). The enhanced activity is primarily attributed to selenium's ability to induce reactive oxygen species (ROS) accumulation in tumor cells, which activates the mitochondrial apoptosis pathway and inhibits tumor cell proliferation and migration. Furthermore, selenized PGPs downregulate the expression of VEGF and PD-1, simultaneously inhibiting angiogenesis and enhancing immune surveillance (32).

#### 2.3.4 Edible-medicinal applications of PGPs

##### 2.3.4.1 Applications in functional foods

PGPs are widely used in functional foods due to their safety and nutritional value: Dairy products: adding 0.3%−0.5% PGPs to yogurt improves antioxidant stability—DPPH scavenging rate remains 75% after 21 days of cold storage (vs. 45% in plain yogurt) (25). Cereal products: PGP-enriched cereal bars (0.4% PGPs) have a sensory score of 8.5/10 (vs. 6.8/10 for plain bars) and exhibit 65% higher antioxidant activity (23). Beverages: UAE-prepared PGPs (0.3% addition) are used in clear drinks with transmittance >90% and DPPH scavenging rate >75% (7).

##### 2.3.4.2 Applications in health foods and pharmaceuticals

Immune-enhancing health foods: PGP chewable tablets (PGPs:maltodextrin:mannitol = 1:2:1) have been approved as “blue hat” health foods in China, with a recommended dose of 1–2 g/day (10). Antitumor adjuvants: sulfated PGPs (100 mg/kg) synergize with cisplatin to reduce tumor volume by 62% (vs. 40% for cisplatin alone) in mice (19). Liver protective supplements: PGP soft capsules (200 mg/capsule) alleviate drug-induced liver injury—ALT levels decrease by 38% in clinical trials (*n* = 50) (11).

##### 2.3.4.3 Applications in cosmetics

PGPs' antioxidant and anti-inflammatory properties make them ideal cosmetic ingredients: anti-aging products: 0.5% PGPs in facial serums reduce skin MDA content by 42% and increase SOD activity by 35% after 4 weeks of use (39). Acne treatments: PGPs inhibit sebum secretion by 30% and reduce inflammatory cytokine (IL-8) levels by 45% in human sebocytes (39).

##### 2.3.4.4 Technical challenges in applications

Poor solubility is a key challenge, as high-molecular-weight PGPs (>50 kDa) have a solubility of < 10 mg/ml in water. Microencapsulation with β-cyclodextrin offers a solution, as it improves the solubility of PGPs to >30 mg/ml (7). Sensory impact: PGPs have a slightly bitter taste. Combining with stevia (0.1%) masks bitterness without affecting bioactivity (40). Stability: PGPs are sensitive to high temperature (>80 °C) and acidic conditions (pH < 3.0). Spray-drying (inlet temperature: 180 °C; outlet temperature: 80 °C) improves thermal stability (12).

#### 2.3.5 Quality control of PGPs

##### 2.3.5.1 Quality indicators

A comprehensive quality control system for PGPs should include the following indicators: purity: >85% (determined by phenol-sulfuric acid method, with glucose as standard) (4). Molecular weight distribution: 6–15 kDa (measured by HPGPC with TSKgel G4000PWxl column) (12). PGPs within this range exhibit the highest bioactivity—molecular weight >50 kDa leads to poor solubility, while < 3 kDa reduces immunomodulatory activity (9). Monosaccharide composition: Ara:Gal:Glc ≈ 1.4:1.0:0.8 (analyzed by HPLC with a Shodex Sugar SP0810 column) (24). Heavy metal residues: Pb < 0.1 mg/kg; Cd < 0.05 mg/kg; As < 0.05 mg/kg (determined by ICP-MS) (23). Microbial limit: total aerobic bacteria < 103 CFU/g; molds and yeasts < 10^2^ CFU/g; no pathogenic bacteria (e.g., *E. coli, Salmonella*) (40).

##### 2.3.5.2 Detection methods and methodology validation

Purity determination: phenol-sulfuric acid method—linear range: 0.02–0.1 mg/ml (*R*^2^ = 0.9998); recovery rate: 95%−105%; RSD < 2% (4). Molecular weight measurement: HPGPC—mobile phase: 0.1 M NaNO_3_; flow rate: 0.8 ml/min; column temperature: 35 °C. The relative standard deviation (RSD) of retention time is < 1.5% (20). Monosaccharide analysis: acid hydrolysis (2 M TFA, 121 °C, 2 h) followed by HPLC—recovery rate: 90%−100%; RSD < 3% (24).

##### 2.3.5.3 Current standards and future recommendations

Currently, no international standard for PGPs exists. The Chinese Pharmacopoeia (2020 Edition) only specifies the identification of platycodin D in *P. grandiflorum* roots, not PGPs (3). We recommend: establishing industry standards: define minimum purity (>85%), molecular weight range (6–15 kDa), and monosaccharide composition for PGPs. Developing rapid detection methods: near-infrared spectroscopy (NIRS) for on-site determination of PGPs content, reducing detection time from 24 h to 10 min (23). Implementing traceability systems: track *P. grandiflorum* origin, extraction/purification processes, and storage conditions to ensure batch consistency.

#### 2.3.6 Limitations

Although PGPs have shown promising potential, there are still several limitations in the current research that need to be acknowledged. Firstly, most of the evidence for bioactivity is based on *in vitro* and animal studies, with a lack of rigorous clinical trials to confirm their efficacy and safety in humans. Secondly, the composition and bioactivity of PGPs can vary greatly depending on the geographical origin, cultivar, and processing methods of *P. grandiflorum*, making it difficult to standardize the quality of the material (41). Finally, industrial scalability remains a challenge due to high costs associated with advanced extraction and purification techniques as well as inherent problems like poor solubility of high molecular weight PGPs which must be addressed before they can be widely used commercially.

## 3 Discussion and future prospects

This review systematically summarizes current research progress on *Platycodon grandiflorum* polysaccharides (PGPs), encompassing extraction and purification methodologies (notably ultrasonic-assisted extraction as an efficient approach), structural characterization techniques, multifunctional bioactivities including immunomodulation, antioxidant capacity, antitumor effects, and hypoglycemic properties with elucidated molecular mechanisms, practical implementations in pharmaceutical formulations and functional food products, as well as ongoing quality control standardization initiatives. Chemical derivatization strategies such as sulfation and selenization demonstrate significant enhancement of PGPs' biological activities, thereby expanding their translational potential in medical therapeutics and nutritional health interventions. However, several critical challenges persist: structure-activity relationships, particularly correlations between branching degree/glycosidic linkage configurations and specific bioactivities like immunomodulation and antitumor effects, remain incompletely elucidated; clinical evidence remains limited with most bioactivity studies conducted *in vitro* or through animal models, coupled with scarcity of clinical trials evaluating long-term safety profiles; production economics present obstacles where purification technologies including ultrasonic-assisted extraction and membrane separation processes increase manufacturing costs by 30%−40%, impeding large-scale industrialization; and product consistency requires improvement due to batch-to-batch quality variations arising from diverse botanical sources and processing methodologies.

Future research priorities should focus on targeted structural engineering approaches—such as β-glucosidase-catalyzed modulation of PGPs' branching architecture and glycosidic linkage patterns to optimize specific bioactivities including antitumor angiogenesis inhibition for cancer therapy—and rigorous clinical trial design implementing randomized, double-blind, placebo-controlled studies to evaluate PGPs' efficacy in immune enhancement for geriatric populations and metabolic disorders like type 2 diabetes mellitus; the study focuses on three key areas: development of green extraction technologies integrating UAE with deep eutectic solvents to achieve cost reductions of 25%−30% while maintaining high yield and purity; multi-omics analysis through integrated transcriptomics, metabolomics, and proteomics to elucidate mechanisms of plant growth promoters, particularly gut microbiota-metabolite-PGPs interactions; and intellectual property protection via proprietary technologies for PGPs modulation and applications, including patentable functional food formulations to facilitate industrialization. This systematic investigation establishes a foundational framework for evidence-based development and industrial implementation of PGPs in health sciences.
